# Assessment of the HScore as a predictor of disease outcome in patients with COVID-19

**DOI:** 10.1186/s12890-021-01706-0

**Published:** 2021-10-29

**Authors:** Mohammadreza Bordbar, Anahita Sanaei Dashti, Ali Amanati, Eslam Shorafa, Yasaman Mansoori, Seyed Javad Dehghani, Hossein Molavi Vardanjani

**Affiliations:** 1 Hematology Research Center, Shiraz University of Medical Sciences, Shiraz, Iran; 2 Professor Alborzi Clinical Microbiology Research Center, Shiraz University of Medical Sciences, Shiraz, Iran; 3 Pediatrics Department, Division of Intensive Care, Medical School, Shiraz University of Medical Sciences, Shiraz, Iran; 4 Student Research Committee, Shiraz University of Medical Sciences, Shiraz, Iran; 5 Shiraz University of Medical Sciences, Shiraz, Iran; 6 Research Center for Traditional Medicine and History of Medicine, School of Medicine, Shiraz University of Medical Sciences, Zand St., Shiraz, Iran

**Keywords:** SARS-coronavirus-2, COVID-19, Cytokine storm, The severity of illness index

## Abstract

Severe coronavirus disease 2019 (COVID-19) accompanies hypercytokinemia, similar to secondary hemophagocytic lymphohistiocytosis (sHLH). We aimed to find if HScore could predict disease severity in COVID-19. HScore was calculated in hospitalized children and adult patients with a proven diagnosis of COVID-19. The need for intensive care unit (ICU), hospital length of stay (LOS), and in-hospital mortality were recorded. The median HScore was 43.0 (IQR 0.0–63.0), which was higher in those who needed ICU care (59.7, 95% CI 46.4–72.7) compared to those admitted to non-ICU medical wards (38.8, 95% CI 32.2–45.4; *P* = 0.003). It was also significantly higher in patients who died of COVID-19 (105.1, 95% CI 53.7–156.5) than individuals who survived (41.5, 95% CI 35.8–47.1; *P* = 0.005). Multivariable logistic regression analysis revealed that higher HScore was associated with a higher risk of ICU admission (adjusted OR = 4.93, 95% CI 1.5–16.17, *P* = 0.008). The risk of death increased by 20% for every ten units increase in HScore (adjusted OR 1.02, 95% CI 1.00–1.04, *P* = 0.009). Time to discharge was statistically longer in high HScore levels than low levels (HR = 0.41, 95% CI 0.24–0.69). HScore is much lower in patients with severe COVID-19 than sHLH. Higher HScore is associated with more ICU admission, more extended hospitalization, and a higher mortality rate. A modified HScore with a new cut-off seems more practical in predicting disease severity in patients with severe COVID-19.

## Introduction

Secondary hemophagocytic lymphohistiocytosis (sHLH) is a life-threatening severe systemic hyperinflammatory syndrome characterized by hypercytokinemia with multi-organ dysfunction. Viral etiologies have been recognized as triggers of sHLH and account for approximately 35% of adult cases [1].

Recent literature by Zhou et al. indicates that severe coronavirus disease 2019 (COVID-19) accompanies by an aggressive inflammatory response known as a cytokine storm [2], that bears similarities to sHLH (including excess production of tumor necrosis factor-α (TNF- α), monocyte chemoattractant protein-1 (MCP1), and interleukin-2 [IL-2]). The probability of sHLH can be predicted by a validated score called HScore [3]. A score higher than 169 was shown to predict the risk of sHLH with 93% sensitivity and 86% specificity [4]. Mehta and colleagues hypothesized that HScore might detect hyperinflammatory states in patients with severe COVID-19. Accordingly, HScore may help to decide which patients may benefit from immune-modulators such as high-dose steroid, interleukin (IL)-1, or IL-6 blockade by anakinra or tocilizumab [5]. However, the use of HScore for COVID- 19 patients has remained questionable by some authors due to limitations regarding body temperature, raised ferritin levels (in early phase ferritin concentrations hardly reach the HScore threshold of 2000 ng/mL), leukopenia (the HScore is unable to distinguish between lymphocytopenia and neutropenia), and the lack of published data on characteristic features [6, 7].

Hypothesizing that the cytokine storm is the principal cause of disease severity in patients with COVID-19, and considering the controversies in the literature regarding the potential of HScore, as an index of cytokine storm, to predict the severity of COVID-19, our study aimed to investigate whether and how HScore is associated with the need for ICU care, hospital length of stay (LOS), and in-hospital mortality in confirmed cases of COVID-19.

## Methods

### Study design

This multi-center, prospective cohort study was conducted in five hospitals dedicated to caring for patients with COVID-19 in Shiraz, Southern Iran. The study population included pediatric and adult patients with a confirmed diagnosis of COVID-19 by reverse transcription-polymerase chain reaction (RT-PCR) on nasopharyngeal swabs. The sampling was done in a subgroup of patients with COVID-19 who needed hospitalization. The need for ICU care was defined as the study’s primary outcome. The study’s secondary outcomes were the length of hospital stay (LOS) in days and in-hospital mortality.

### Participants

Eligible adults and pediatric patients were consecutively enrolled in the study from July 22 to September 21, 2020, if they consent to participate. A sample size of 196 patients with an allocation ratio of 2.5 was calculated, assuming an observed ratio of 1:2–3 for patients who need ICU care versus patients without the need for ICU care in our setting. The attending physicians and the intensivists decided which patients should be transferred to ICU based on their clinical judgment. We assumed that the need for ICU care in patients with cytokine storm is more than patients without cytokine storm [8]. A type I statistical error of 0.025 and statistical power of 90% was assumed.

### Data collection

The demographic data, including age, gender, COVID-19 signs and symptoms, duration of symptoms before admission, and comorbid illnesses, were recorded at the time of hospital admission. The presence of hepatosplenomegaly was documented either by physical examination or ultrasonography. Given the high-intensity condition and to protect hospital staff, it was decided to rely on bedside physical examination in most patients. If ultrasonography was performed for other purposes, the results were taken into consideration. Laboratory data including complete blood count (CBC), liver function test (LFT), serum triglyceride (TG), ferritin, fibrinogen, D-dimer, glucose, sodium (Na), potassium (K), blood urea nitrogen (BUN), creatinine (Cr), calcium (Ca), Phosphorus (P), erythrocyte sedimentation rate (ESR), C-reactive protein (CRP), lactic dehydrogenase (LDH), creatine phosphokinase (CPK), prothrombin time (PT), activated partial thromboplastin time (aPTT), procalcitonin, and troponin levels were measured in the fasting state upon admission. We did not perform bone marrow aspiration because of ethical considerations in these high-risk, critically ill patients. Therefore, a zero score was recorded for those items with no available data, such as hemophagocytosis in bone marrow smear. A trained physician interviewed the patients and collected the data in the data entry form.

### Statistical analysis

Statistical analysis was done using Stata software released 11. Mean and standard deviation (SD), median and inter-quartile range (IQR), and frequencies were used for data description. Given the low number of an HScore of higher than 169 among the study participants, as a practical solution, participants were categorized into three relatively equal groups (Low, Moderate, and High HScore), based on tertiles of the original value of patients’ HScore. Chi-square and two-sample Wilcoxon Rank-Sum tests were used for univariate analyses to identify associated factors with the need for ICU care and in-hospital mortality. Two multivariable binary logistic regression models were fitted to determine the independent association of HScore with the need for ICU care (Yes/No) and in-hospital mortality (Yes/No). Data on LOS was described using the estimated median time to discharge. Log-rank test and Kaplan–Meier survival curves were used to identify univariate determinants of LOS. Proportional hazard Cox regression modeling was applied to identify the independent factors associated with LOS. All multivariable regression modeling was done applying the backward elimination technique. A P-value of 0.05 or less was considered a significant level.

### Ethics and consent to participate

The study was approved by the Research Ethics Committee of Shiraz University of Medical Sciences. The project was in line with the ethical principles and the national norms and standards for conducting Medical Research in Iran with approval ID IR.SUMS.REC.1399.165 on 2020-04-26 [9]. All individuals (or their parents) in the study population were informed about the current study, with written consent obtained before enrolment in the present study.

## Results

**Fig. 1:**
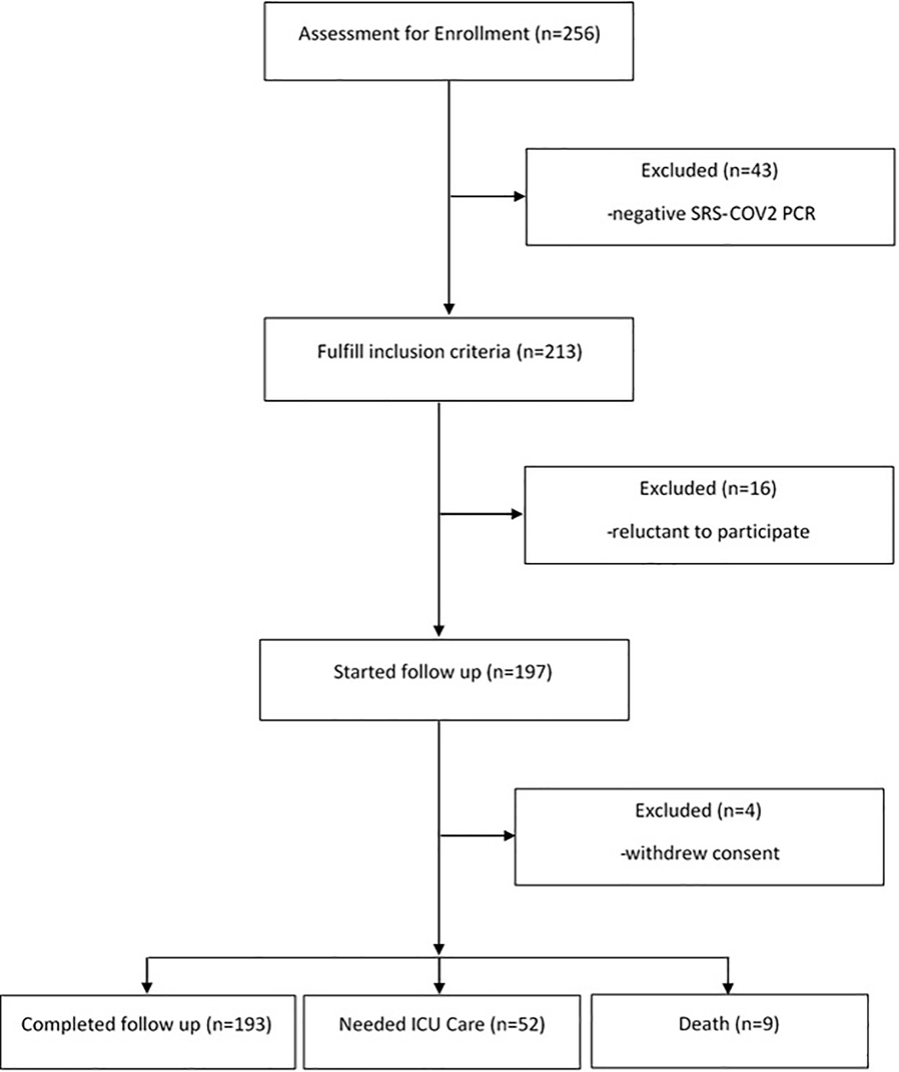
Study flowchart

One hundred ninety-three patients (49.2% females) with a confirmed diagnosis of COVID-19 and a median age of 47.0 ± 23.7 years (range 1.5–93 years) completed the study. The study included 35 (18.1%) children below the age of 18 years. In addition, 52 (26.94%) patients needed ICU care (Fig. 1).

The median hospital LOS was seven days (IQR 5–11.5). It was ten days (IQR 6.25 to 15) in the subgroup that needed ICU admission. We encountered nine death events (equal to a mortality rate of 4.66% with a 95% CI of 2.42–8.77%). Out of them, 8 (88.9%) occurred among patients admitted to ICU wards. The most common presenting symptoms were lower respiratory symptoms, including cough, sputum production, chest pain, and shortness of breath (63%), followed by constitutional symptoms including fatigue, myalgia, generalized body pain, headache, loss of appetite (40%), and gastrointestinal symptoms including abdominal pain, nausea, vomiting, diarrhea (18%). The background characteristics of the study cohort are summarized in Table 1.

**Table 1:** Demographic and clinical presentation of patients with COVID-19

	ICU patients (n = 52)		Non-ICU patients (n = 141)		P*
	n(%)	HScore Mean (SD)	n(%)	HScore Mean (SD)	
Gender					
Male	25 (48.1)	58.9 (39.7)	73 (51.8)	39.3 (41.0)	0.040
Female	27 (51.9)	60.4 (53.2)	68 (48.2)	38.3 (38.8)	0.027
*Age group*					
Younger than 19 Years	18 (51.4)	89.7 (51.2)	17 (48.6)	110.5 (42.1)	0.621
Older than 18 Years	34 (21.5)	43.8 (35.8)	124 (78.5)	29.0 (27.6)	0.033
Obesity					
Yes	10 (19.2)	43.5 (22.9)	11 (7.8)	28.6 (22.1)	0.144
No	42 (80.8)	63.6 (50.3)	130 (92.2)	39.7 (40.8)	0.002
Overweight					
Yes	19 (36.5)	51.5 (46.8)	84 (59.6)	43.5 (43.6)	0.477
No	33 (63.5)	64.4 (46.8)	57 (40.4)	31.8 (32.3)	< 0.001
Smoking					
Yes	2 (3.9)	31.5 (44.5)	7 (5.0)	18.5 (19.8)	0.538
No	51 (96.1)	60.8 (46.9)	134 (95.0)	39.8 (40.3)	0.003
*Symptoms*					
Fever					
Yes	20 (38.5)	65.3 (38.7)	38 (27.0)	54.7 (53.8)	0.438
No	32 (61.5)	56.2 (51.5)	103 (73.0)	32.9 (32.5)	0.002
Chills					
Yes	47 (90.4)	62.5 (47.9)	111 (78.7)	42.1 (41.6)	0.008
No	5 (9.6)	33.8 (24.5)	30 (21.3)	26.5 (29.3)	0.601
Sore throat					
Yes	0	–	8 (5.7)	98.8 (70.3)	–
No	52 (100)	59.7 (46.8)	133 (94.3)	35.2 (34.4)	< 0.001
Dyspnea					
Yes	28 (53.8)	57.9 (53.7)	63 (44.7)	27.1 (30.2)	0.001
No	24 (46.2)	61.8 (38.1)	78 (55.3)	48.2 (38.3)	0.175
Loss of appetite					
Yes	1 (1.9)	146 (–)	20 (14.2)	85.5 (57.2)	–
No	51 (98.1)	58.0 (45.6)	121 (85.8)	31.3 (30.0)	< 0.001
Abdominal pain					
Yes	5 (9.6)	117.4 (78.0)	11 (7.8)	97.4 (11.0)	0.611
No	47 (90.4)	53.6 (38.7)	130 (92.2)	33.8 (32.2)	< 0.001
*Length of hospital stay (days)*					
Mean (SD)	52 (100)	14.40 (7.6)	141 (100)	6.14 (4.9)	< 0.001
Median (IQR)	52 (100)	14.5 (9.0, 18.5)	141 (100)	7.0 (2.0, 8.0)	< 0.001
*Comorbidities*					
Hypertension					
Yes	14 (26.9)	43.8 (29.2)	39 (27.7)	27.3 (26.5)	0.057
No	38 (73.1)	65.6 (50.9)	102 (72.3)	43.2 (43.1)	0.010
Diabetes mellitus					
Yes	13 (25.0)	43.7 (31.1)	32 (22.7)	30.5 (26.7)	0.158
No	39 (75.0)	65.1 (50.2)	109 (77.3)	41.2 (42.6)	0.005
Cardiovascular diseases					
Yes	8 (14.4)	33.1 (32.5)	23 (16.3)	28.7 (27.3)	0.709
No	44 (84.6)	64.5 (47.6)	118 (83.7)	40.8 (41.6)	0.002
Respiratory diseases					
Yes	2 (3.9)	15 (21.2)	10 (7.1)	43.2 (33.9)	0.294
No	50 (96.1)	61.5 (46.7)	131 (92.9)	38.5 (40.3)	0.001
Hypothyroidism					
Yes	1 (1.9)	19 (-)	6 (4.3)	16.7 (24.5)	–
No	51 (98.1)	60.5 (46.9)	135 (95.7)	39.8 (40.1)	0.003
Neuropsychiatric disorders					
Yes	5 (9.6)	92.6 (72.6)	16 (11.4)	26.9 (27.6)	0.006
No	47 (90.4)	56.2 (42.9)	125 (88.7)	40.3 (40.9)	0.026
In-hospital mortality					
Yes	8 (15.4)	105.1 (71.5)	1 (0.7)	105 (–)	–
No	44 (84.6)	51.4 (36.2)	140 (99.3)	38.3 (39.5)	0.052

*SD* standard deviation, *IQR* inter-quartile range, *ICU* intensive care unit

*P value compares patients with and without ICU care in terms of the mean of HScore

The median HScore was 43.0 (IQR: 0.0 to 63.0). The mean HScore was higher in those who needed ICU care (59.7, 95% CI 46.4–72.7) than those admitted to non-ICU medical wards (38.8, 95% CI: 32.2 to 45.4; *P* = 0.003). The mean HScore was also significantly higher in patients who died of COVID-19 (105.1, 95% CI 53.7–156.5) than individuals who survived (41.5, 95% CI 35.8–47.1; *P* = 0.005). Regarding signs and symptoms, patients with fever, sore throat, loss of appetite, and abdominal pain as their present illness showed higher HScore compared to those without these symptoms (Table 1).

**Fig. 2:**
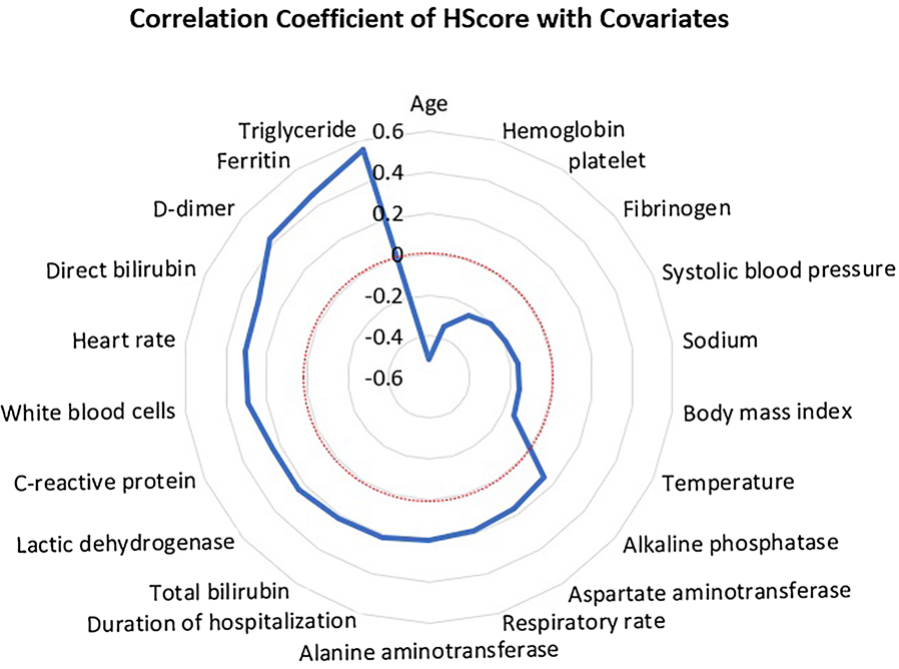
Correlation coefficients of HScore with other continuous variables in Covid-19 patients (radar graph). The dashed red line represents a zero-correlation coefficient. Coefficients with a statistically significant value are presented in the graph

The correlation coefficients of continuous variables with HScore are also shown in Fig. 2. Among the measured variables, age, hemoglobin, platelet, sodium, fibrinogen, systolic blood pressure, body mass index, and temperature were negatively correlated with HScore. On the other hand, triglyceride, ferritin, D-dimer, total and direct bilirubin, AST, ALT, ALP, white blood cell counts, CRP, LDH, heart rate, respiratory rate, and duration of hospitalization were positively correlated with HScore (Fig. 2).

The mean HScore (95% CI) in low (n = 80), moderate (n = 73), and high (n = 40) HScore categories were as follow: Low HScore (7.4, 95% CI 5.3–9.4), moderate HScore (49.2, 95% CI 46.4–52.0), and high HScore (109.8, 95% CI 98.7–120.9). Forty-nine patients (25.4%) showed a zero HScore. The maximum score detected was 202. Only four patients had an HScore higher than the cut-off value of 169. Among them, two patients needed ICU admission, and both of them died. HScore in these patients was correlated with mortality (*P* = 0.01) but not with hospital LOS (*P* = 0.41) or ICU need (*P* = 0.57).

**Fig. 3:**
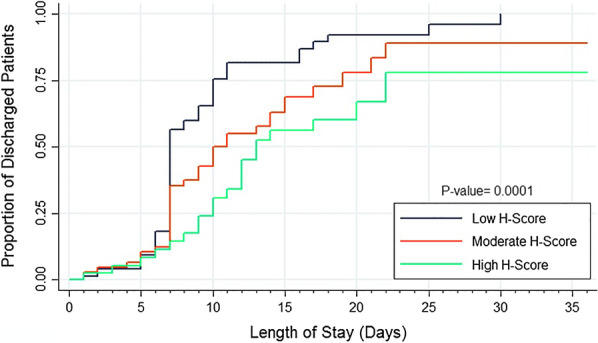
Kaplan–Meier survival curve of hospital length of stay in three categories of HScore

Table 2 demonstrates the multivariable logistic regression analysis of independent factors associated with the risk of ICU admission, mortality, and duration of hospitalization. According to multivariable models, patients with higher HScore had almost five times the risk of ICU admission (adjusted OR = 4.93, 95% CI 1.5–16.17, *P* = 0.008) compared to those in the lower HScore level. The risk of death increased by 20% for every ten units increase in HScore (adjusted OR 1.02, 95% CI 1.00–1.04, *P* = 0.009). Moreover, patients who needed ICU care were at risk of death more than 66 times than patients admitted to medical non-ICU wards (adjusted OR = 66.06, 95% CI 3.76–1160.45, *P* = 0.004). Time to discharge was statistically longer in high (HR = 0.41, 95% CI 0.24–0.69) and moderate (HR = 0.61, 95% CI 0.39–0.94) HScore levels compared to low level (Table 2; Fig. 3).

**Table 2:** Multivariable logistic regression of independent factors associated with the risk of ICU admission, mortality, and duration of hospitalization

Variables	Crude effect measure (95% CI)	*P* value	Adjusted effect measure (95% CI)	*P* value
	Crude OR (95% CI)		Adjusted OR (95% CI)	
*ICU admission (as outcome)*				
HScore				
Moderate	2.95 (1.35–6.44)	0.007	4.58 (1.78–11.79)	0.002
High	3.4 (1.40–8.22)	0.007	4.93 (1.50–16.17)	0.008
Overweight	0.39 (0.20–o.75)	0.005	0.35 (0.14–0.82)	0.017
Diastolic Blood pressure	0.95 (0.93–0.98)	0.002	0.96 (0.92–0.99)	0.046
Loss of appetite	0.11 (0.01–0.9)	0.040	0.20 (0.001–0.25)	0.002
Fatigue	1.04 (0.50–2.19)	0.899	5.25 (1.67–16.48)	0.004
Lymphocyte	0.91 (0.87–0.95)	< 0.001	0.90 (0.86–0.94)	< 0.001
Hemoglobin	0.81 (0.70–0.93)	0.003	0.73 (0.60–0.88)	0.001
Age	0.99 (0.98, 1.00)	0.187	0.99 (0.97, 1.01)	0.399
Gender (Ref.: female)	0.86 (0.46, 1.63)	0.649	0.48 (0.20, 1.14)	0.097
*Mortality (as outcome)*				
HScore	1.02 (1.01–1.03)	< 0.001	1.02 (1.00–1.04)	0.009
ICU admission	25.5 (3.1–209.2)	0.003	66.06 (3.8–1160.5)	0.004
Systolic Blood pressure	0.94 (0.91–0.98)	0.006	0.93 (0.88–0.98)	0.009
Potassium	0.19 (0.05–0.72)	0.015	0.11 (0.02–0.63)	0.013
Age	0.98 (0.95, 1.00)	0.098	1.06 (0.99, 1.12)	0.083
Gender (Ref.: female)	0.47 (0.11, 1.92)	0.294	0.49 (0.06, 3.87)	0.497

Variables	Crude effect measure (95% CI)	*P* value	Adjusted effect measure (95% CI)	*P* value
	Crude HR (95% CI)		Adjusted HR (95% CI)	
*Discharge from hospital (as outcome)*				
HScore*				
Moderate	0.61 (0.39–0.94)	0.024	0.60 (0.37–0.97)	0.036
High	0.41 (0.24–0.69)	0.001	0.42 (0.22–0.78)	0.007
Cardiovascular disease	0.67 (0.38–1.21)	0.185	0.40 (0.21- 0.78)	0.007
Respiratory disease	4.24 (2.02–8.90)	< 0.001	5.47 (2.39–12.52)	< 0.001
Chronic kidney disease	0.52 (0.13–2.14)	0.362	11.09 (1.38–89.10)	0.024
Respiratory status	1.40 (1.09–1.81)	0.009	1.34 (1.03–1.74)	0.027
Oxygen therapy	0.43 (0.25–0.73)	0.002	0.22 (0.11- 0.45)	< 0.001
Pneumonia	5.26 (2.53–10.94)	< 0.001	4.56 (1.99–10.46)	< 0.001
Septic shock	0.42 (0.20–0.87)	0.020	0.12 (0.04–0.34)	< 0.001
White blood cell counts	0.999 (0.993–1.004)	0.719	1.006 (1.00–1.01)	0.042
Red blood cell counts	1.82 (1.40–2,36)	< 0.001	1.59 (1.16–2.18)	0.004
Lymphocyte	1.03 (1.02–1.04)	< 0.001	1.03 (1.01–1.04)	0.001
Aspartate aminotransferase	0.996 (0.991–1.001)	0.178	1.006 (1.00–1.01)	0.023
Alanine aminotransferase	0.995 (0.989–1.001)	0.178	0.98 (0.97–0.99)	0.011
Phosphorus	0.83 (0.74–0.93)	0.002	0.82 (0.69–0.98)	0.027
Age	0.99 (0.98, 1.00)	0.110	0.99 (0.98, 1.00)	0.419
Gender (Ref.: female)	0.79 (0.54, 1.15)	0.217	1.13 (0.73, 1.74)	0.584

*Moderate and high HScore were compared with low HScore as the reference group

## Discussion

Our study focused on the utility of HScore as a predictive index of disease severity in patients with COVID-19. We found that higher HScore is associated with a higher chance of ICU admission, increased risk of mortality, and longer length of hospital stay in patients with a confirmed diagnosis of COVID-19. However, almost all of our patients had a score much lower than the proposed cut-off of 169. Even those who died of their illness (9 patients) had a mean HScore of 105.1.

The limitations of HScore in predicting severe cases of COVID-19 were previously emphasized by other investigators [7, 10–13]. Most critical cases of COVID-19 did not reach the proposed cut-off of 169 in sHLH. The following features in the novel Coronavirus disease may explain the differences with sHLH resulting in lower HScore. First, patients with severe COVID-19 usually present with leukocytosis and lymphopenia and not leukopenia. Second, hepatosplenomegaly is not a common finding in COVID-19. Third, the peak temperature, which gets a high score in HScore, does not seem to correlate with disease severity in COVID-19. Fourth, low fibrinogen levels are a common finding in sHLH, while patients with COVID-19 usually have normal or high fibrinogen and D-dimer levels. Fifth, hypertriglyceridemia as a result of lipoprotein lipase inhibition by TNF-α is not usually reported in COVID-19. Finally, though serum ferritin is increased in viral infections, including SARS-Cov2 infection, it rarely raises more than 2000 ng/ml except in the final stages of the disease [6, 7].

Although the severity of systemic inflammation in SARS-Cov2 shares similarities with MAS or sHLH, the main difference is in the immunopathology of COVID-19 pneumonia. Patients with severe SARS-Cov2 usually experience a localized cytokine storm in the lung tissue resulting in pulmonary intravascular coagulopathy rather than disseminated intravascular coagulation. Therefore, systemic manifestations such as hepatosplenomegaly, pancytopenia, low fibrinogen, and liver dysfunction typical of sHLH are less evident in COVID-19 unless in the later stages of the disease [14]. Besides, lytic death of epithelial and endothelial cells in the lung following COVID-19 causes a massive release of IL1-α, which is mainly involved in local microenvironment inflammation. Consequently, the activation of infiltrating macrophages expressing IL-1R1 leads to overproduction of IL1-β, which takes the inflammation beyond the local level culminating in widespread systemic inflammation [15].

Our patients had a median HScore of 43.0. As we did not perform bone marrow aspiration and biopsy in our patients, a zero score was assigned to this item. There is not enough evidence of what proportion of patients with COVID-19 will show hemophagocytosis in reticuloendothelial systems. It was reported in an autopsy series in fatal cases of COVID-19 that 10 out of 15 patients showed evidence of erythrophagocytosis in bone marrow smears [16]. Another autopsy report in four COVID-19 patients with diffuse alveolar damage proved lymphophagocytosis in pulmonary lymph nodes in 3 patients [17]. Although hemophagocytosis is not specific for sHLH and is seen in other conditions such as blood transfusion, malignancy, or autoimmune disorders, it may designate severe SARS-Cov2 infections leading to a fatal outcome. Assuming that all the patients had evidence of hemophagocytosis in bone marrow smears, the median HScore will be 78.0. In that case, up to 9 patients would have a score higher than 169, which is still too low.

Despite the mentioned limitations, higher HScore was correlated with a more extended hospital stay, higher rate of ICU admission, and higher mortality rate in our cohort of COVID-19. Patients with higher HScore had an almost 60% lower chance of being discharged from the hospital than patients with lower HScore (HR = 0.42). In addition, they needed ICU care almost five times that of patients with lower HScore (OR = 4.93). The risk of mortality was also increased by 20% for every ten units rise in HScore (OR 1.02). These figures may support this hypothesis that HScore can be relied on as an index of disease severity in patients with COVID-19. However, a new cut-off should be introduced rather than the traditional cut-off of 169, which is most helpful in sHLH [18]. Besides, a modified HScore with omitting some parameters such as hemophagocytosis in bone marrow smear and possibly adding other variables related to COVID-19 severity such as D-dimer and chest CT scan findings seems more practical in the setting of COVID-19. It may help distinguish mild and moderate hyper inflammation from acute HLH-like syndromes, presenting with multi-organ failure [19].

Some researchers successfully treated their patients with severe COVID-19 and high HScore (≥ 169) with immunomodulatory agents such as anakinra and tocilizumab [20–24]. Given that such a high score is rarely encountered in SARS-Cov2 infection, we may consider treating patients with cytokine release syndrome using IL-1 blockers even with lower HScore. The exact cut-off for such a decision needs to be defined. It was recently reported that IL-1 inhibition, but not IL-6 inhibition, was associated with a 55% reduction in mortality risk in patients with severe COVID-19 who presented with respiratory insufficiency and hyper inflammation. The effect was more pronounced in a subgroup of patients with lower LDH than those with higher LDH values [25].

Some clinical symptoms such as fever, sore throat, loss of appetite, and abdominal pain were associated with higher HScore. Additionally, younger age, lower temperature, systolic blood pressure, and higher heart and respiratory rates on admission were associated with higher HScore. Such an association was not previously described elsewhere. It is expected that higher temperature may be associated with higher HScore in sHLH since it will get a higher score in the final calculation. However, this is not the case in COVID-19, and the higher temperature does not necessarily mean a more severe infection. We observed that lower temperature was correlated with higher HScore. Additionally, a subgroup of patients without fever admitted to the ICU had higher HScore than non-ICU patients (Table 1). It seems that the height of temperature is not an important determinant of disease severity in patients with COVID-19.

Among different laboratory values, anemia, thrombocytopenia, hypofibrinogenemia, and hyponatremia were correlated with higher HScore. Similarly, patients with leukocytosis, hypertriglyceridemia, hyperferritinemia, high D-dimer, CRP, LDH, and abnormal liver function tests showed higher HScore. A simple explanation could be that they may indicate a more severe illness. Moreover, some parameters such as cytopenia, lower fibrinogen, and higher serum ferritin, triglyceride, and liver enzymes are directly correlated with higher HScore, since they are included in calculating the total score. However, the causality cannot be assured, and the confounding effect of other covariates should be considered.

The study has some limitations. Although our sample size was adequate to show if the need for ICU care is associated with HScore, because of the rarity of cytokine storm among COVID-19 patients, further studies with a larger sample size are needed to determine the most appropriate cut-off point of HScore to predict prognosis of COVID-19. In addition, we faced a few deaths in our cohort, which may skew the results and made us cautious about the outcome assessment. Besides, larger sample size may provide opportunities to investigate the correlation of disease severity and cytokine storm among patients within different subgroups, such as patients with loss of appetite or sore throat or patients with different rare comorbidities.

In conclusion, HScore is usually much lower in patients with severe COVID-19 than sHLH. Thus, it is not a sensitive marker to predict disease severity in SARS-Cov2 infection. However, higher HScore is associated with more extended hospitalization, higher ICU admission, and a higher mortality rate. Therefore, a modified HScore with a new cut-off should be defined.

## Data Availability

The datasets used and analyzed during the current study are available from the corresponding authors on reasonable request.
